# Building a performance measurement framework for telephone triage services in Finland: a consensus-making study based on nominal group technique

**DOI:** 10.1186/s13049-024-01243-9

**Published:** 2024-08-13

**Authors:** Hanna Vainio, Leena Soininen, Paulus Torkki

**Affiliations:** 1 Department of Public Health, Faculty of Medicine, University of Helsinki, Helsinki, Finland; 2 DigiFinland Ltd., University of Helsinki, Helsinki, Finland

**Keywords:** Emergency care, Telephone triage, Performance measurement

## Abstract

**Background:**

There has been a significant expansion in the measurement of healthcare system performance. However, there is a lack of a comprehensive performance measurement framework to assess the effects of telephone triage services on the urgent care system. The aim of our Delphi study was to construct and validate a performance measurement framework designed explicitly for telephone triage services.

**Methods:**

This study was conducted in Finland with a group of eight experienced senior physicians from the country's 20 largest joint emergency departments, serving over 90% of the population for urgent care. The Nominal Group Technique (NGT) was utilised to achieve consensus on measuring telephone triage performance. Initially, performance indicators (PIs) were identified through Delphi method rounds from December 10th to December 27th, 2021, with eight experts participating, and from December 29th, 2021, to January 23rd, 2022, where five of these experts responded. NGT further deepened these themes and perspectives, aiding in the development of a comprehensive performance measurement framework. The final framework validation began with an initial round from February 13th to March 3rd, 2022, receiving five responses. Due to the limited number of responses, an additional validation round was conducted from October 29th to November 7th, 2023, resulting in two more responses, increasing the total number of respondents in the validation phase to seven.

**Results:**

The study identified a strong desire among professionals to implement a uniform framework for measuring telephone triage performance. The finalised framework evaluates telephone triage across five dimensions: service accessibility, patient experience, quality and safety, process outcome, and cost per case. Eight specific PIs were established, including call response metrics, service utility, follow-up care type and distribution, ICPC-2 classified encounter reasons, patient compliance with follow-up care, medical history review during assessment, and service cost per call.

**Conclusions:**

This study validated a performance measurement framework for telephone triage services, utilising existing literature and the NGT method. The framework includes five key dimensions: patient experience, quality and safety, outcome of the telephone triage process, cost per case, and eight PIs. It offers a structured and comprehensive approach to measuring the overall performance of telephone triage services, enhancing our ability to evaluate these services effectively.

**Supplementary Information:**

The online version contains supplementary material available at 10.1186/s13049-024-01243-9.

## Background

To address increasing demand and enhance future healthcare system efficiency, many countries have established a centralised Medical Helpline 116,117 (MH) telephone triage services. Despite these efforts, Emergency department (ED) overcrowding remains a critical issue, leading to prolonged patient stays, higher inpatient mortality rates, and escalating costs [1–4]. The fundamental aim of many healthcare systems is to enhance their organisational structures in terms of both service quality and efficiency, ensuring optimal utilisation of their resources [5]. Consequently, healthcare organisations have been developing and implementing performance indicators (PIs) to measure and manage system effectiveness, efficiency, equity, and quality [6].

The development of a performance measurement framework for telephone triage could be compromised if sufficient care is not taken in defining, selecting, and prioritising indicators [7]. It is crucial to ensure that the indicators are not only measurable but also maintain the validity and relevance of the framework. The validity of a measurement framework depends on constructing an evidence-based rationale that demonstrates how accurately the framework measures what it is intended to measure [8].

Currently, there is no reliable and comprehensive framework in place to assess the overall performance of telephone triage services. In our preceding scoping review, we found that most studies evaluated the performance of telephone triage services using indicators related to health outcomes, patient experiences, or other limited perspectives [9]. There was no established framework that encompassed all performance dimensions to measure overall performance. Given the absence of a standardised or universally accepted method for assessing performance in telephone triage, validating the indicators that will be incorporated into the framework is imperative.

Identifying appropriate indicators is a complex task, as they are linked to performance factors still associated with the operational processes of the service. Therefore, recognising PIs necessitates considering the opinions and involvement of stakeholders in the decision-making process [10]. This study aims to achieve consensus on the indicators to be used in the measurement framework for telephone triage performance, utilising the nominal group technique (NGT).

## Methods

### Design

The aim of our Delphi study was to construct and validate a performance measurement framework specifically designed for telephone triage services. We employed the Delphi method to identify and filter the most crucial PIs for our measurement framework. Concurrently, we used the three-round NGT to establish consensus among experts.

The Delphi method facilitates structured expert opinion integration, capitalising on its strengths in eliciting informed inputs and fostering consensus-building. Chosen for its robust consensus-building capability, the NGT method ensures balanced participation from all group members, making it particularly effective in scenarios necessitating decision-making, complex problem-solving, prioritisation, and consensus achievement. [11, 12]. The NGT method has found applications in research aimed at generating ideas, solving problems, and establishing priorities [13, 14]. Additionally, the technique is valued for its rapid implementation, transparent process, inclusivity, and ease of replication [14–16].

We combined NGT-led group discussions with a digital survey. The digital survey was particularly useful given the geographical dispersion of NGT group participants, facilitating a time-efficient and inductive exploration of the subject, free from external bias [13, 14]. The NGT process unfolds in two main phases: an initial ranking, where participants evaluate the PIs using a Likert scale, and a subsequent re-ranking, allowing participants to revise their initial evaluations based on insights from a secondary survey [12].

### Setting

When illness symptoms appear, patients can reach out to outpatient healthcare professionals at their local health centre during weekdays for non-urgent conditions. Since 2018, a 24/7 Medical helpline 116,117 has been available in Finland. The MH is accessible throughout Finland, with the exception of Lapland and the Åland Islands. Wellbeing services counties are responsible for organising the service in their area.

After hours, on weekends, and on public holidays, patients are advised to call MH before going to the ED. In case of an emergency, critical illness, or injury, patients need to call the emergency number 112. At the MH, nurses conduct telephone triage using a protocol that follows the national principles of urgent care coordinated by the Ministry of Social Affairs and Health. Ilkka [17] This protocol includes a six-tier urgency classification system. The nurses guide patients to the most appropriate care based on their needs or, in an emergency, forward the call to 112. Calls are documented in the electronic health record (EHR) using the ISBAR protocol. The acronym ISBAR stands for identify, situation, background, assessment, and recommendation [18]. Reasons for encounters are documented using the ICPC-2 classification [19]. Nationally, it is required to document at least the patient's need for care, medical history, status, and plan, i.e., the outcome of the triage. Despite recommendations for call documentation, there is significant variation in the quality and comprehensiveness of documentation among professionals [20, 21].

### Participants

The participant experts in our study were chosen through purposeful sampling. This method was strategically employed to engage experts with in-depth knowledge and vast experience in telephone triage, thereby ensuring the inclusion of information-rich cases. The primary aim of this sampling technique was to facilitate a comparative analysis of expert opinions, allowing us to discern both similarities and differences in their perspectives and insights [22, 23]. The NGT group consisted of chief physicians from all 20 central hospitals in Finland, representing the full geographical spread of emergency services across the country. Each participant specialised in Emergency medicine and/or Anesthesiology and intensive care medicine. Additionally, they were responsible for the operations of the MH, as well as both primary and specialised care ED services within their respective regions. Each respondent also held a leadership position, with oversight and reporting responsibilities for the entirety of urgent care services. This deliberate selection of experts was essential to identify potential regional variations and achieve consensus.

### Data collection

Ten experts participated in the NGT group activities. After agreeing to participate, experts received an email link to access the survey, accompanied by three reminder emails for each round. To accommodate participants' schedules, we extended response deadlines by 11 days for the second round and by one month for the third. Questionnaires were distributed via the Webropol© platform, with the introductory page of each round providing detailed instructions and guidance on completing the questionnaire. Following each round, experts received a summary of the results from the preceding round. This iterative process aimed to achieve the highest possible level of consensus and to support participants' thinking towards the next round.

### First round

While the first round of a classical Delphi process is typically unstructured, we chose to structure this phase based on our understanding of essential indicators for the framework, thereby optimising professional time, as suggested by Rowe et al. (1991). In this round, our expert panel evaluated 28 PIs organised into five distinct performance dimensions. Each PI was rated on a 10-point Likert scale to assess its importance and relevance. Our Delphi process focused on validating these PIs while ensuring their balanced distribution across the framework. To maintain clarity and simplicity, we restricted the number of PIs to two per performance dimension. The decision was made to build a balanced measurement framework, which is common in measurement frameworks for management (e.g. Balanced Scorecard, Quadruple Aim). We also aimed to enhance its practical application in management by choosing a limited number of measures and thus reducing information overload [24]. At the end of the first round, we selected two PIs from each dimension that received the highest scores to proceed to the next round. In cases where two PIs received identical scores, we chose the indicator that had a higher score in terms of relevance for inclusion.

### Second round

The duration for submitting responses in the second round was extended from December 29th, 2021, to January 12th, 2022. Consequently, due to the low rate of response, the deadline was further extended to January 23rd, 2022. Additionally, three reminder emails were dispatched. In this round, respondents appraised the accuracy and sensitivity of the PIs using the same 10-point scale. They assessed how well each indicator represented its assigned performance dimension, with scores below seven prompting suggestions for more accurate alternatives.

### Workshop

Following the second round, we conducted a workshop with the authors of the study to analyse the responses and refine the framework for the final round. In particular, we scrutinised PIs that received a rating of less than 7 from any evaluation perspective, seeking alternative indicators based on open-ended responses. The primary objective was to enhance the framework's effectiveness in advancing the development of telephone triage services and to maximise its strategic value in both short-term and long-term scenarios.

### Third round

**Fig. 1:**
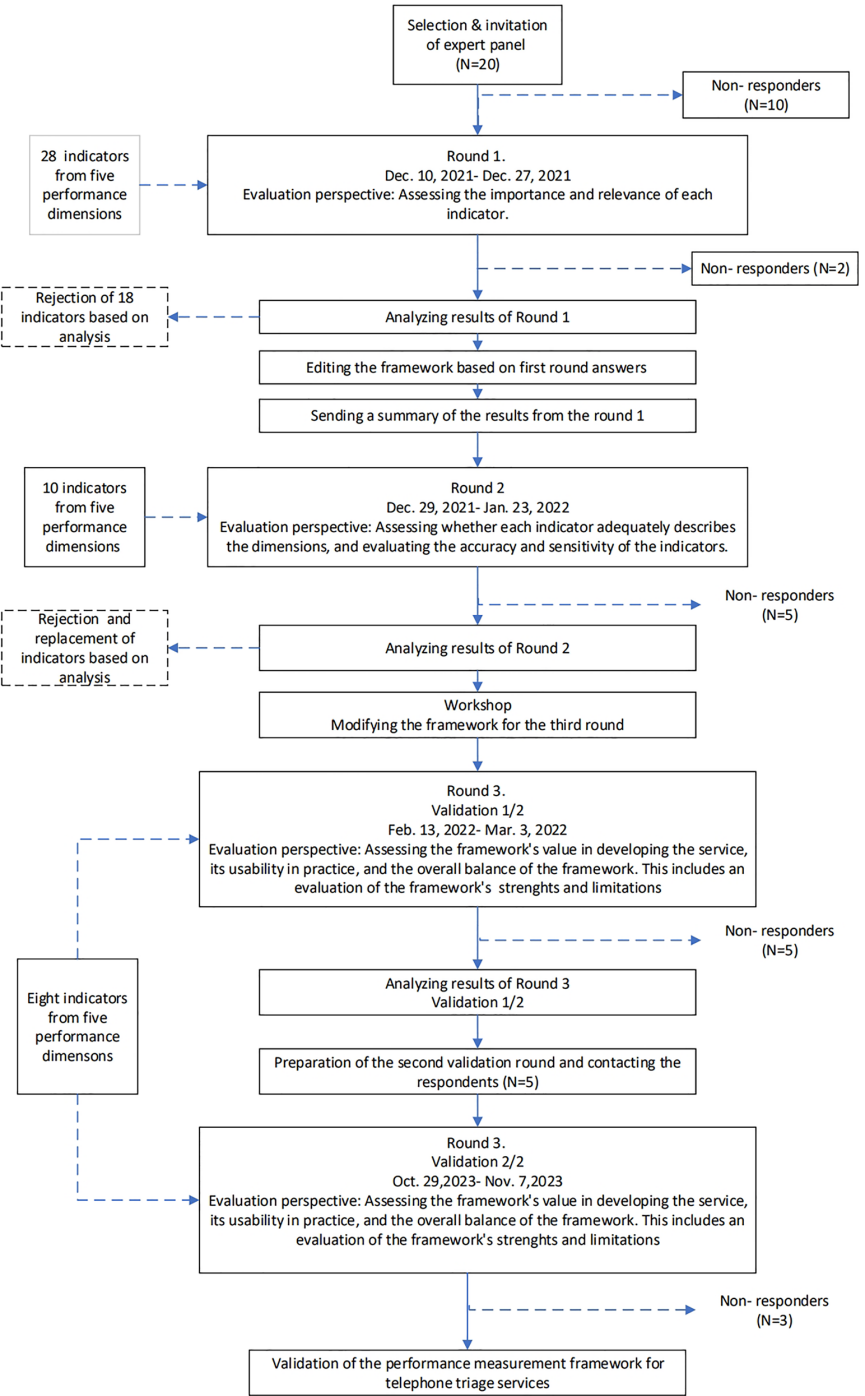
A Flowchart of the research process

The response period for the third round was set from February 13th, 2022, to March 3rd, 2022. In response to the low response rates observed during the first three rounds and to further confirm the utility of our framework, a final performance framework validation round was conducted from October 29th, 2023, to November 7th, 2023. The final evaluation in this round included assessing the framework's contribution to the development of telephone triage services, its utility in aiding both short-term and long-term strategic development, and its overall relevance. Crucially, participants also evaluated the balance of the framework, providing insights into its strengths and limitations and offering open-ended feedback. Upon completion of each round, we entered the data into MS Excel© for analysis. We conducted a descriptive analysis, which included calculating frequency, percentage agreement, mean (indicator scores), and median. A flowchart (Fig. 1) provides an overview of the research process.

### Ethical considerations

Members of the Delphi panel were informed in their invitation email and related materials that they were free to withdraw from the study at any point. Their agreement to participate was confirmed via email, where we emphasised the entirely voluntary nature of their participation. To ensure confidentiality, the names and contact details of the participating professionals were securely stored in a restricted-access location, separate from the main data repository. Due to the study's nature, obtaining institutional review board approval was not required. The University of Helsinki, Finland, coordinates the study. The study adheres to the ethical principles outlined in the Helsinki Declaration.

## Results

Of the 20 experts invited, 10 agreed to participate in this e-Delphi study. The expert panel consisted of senior physicians from Finnish joint emergency departments, who were responsible for providing 24/7 emergency services, including MH services, in their respective areas. In round 1, eight experts completed the questionnaire, while two did not respond. In rounds 2 and 3, five experts responded to the questionnaire. Due to the limited number of responses, a subsequent validation round was conducted, resulting in two additional responses. This increased the total number of respondents in the framework validation phase to seven.

### Round one

The initial round one questionnaire presented to the expert panel featured 28 proposed individual PIs distributed across five distinct performance dimensions (Table 1). Participants were tasked with evaluating the importance and relevance of each PI using a numerical Likert scale. On this scale, 1 represented 'no support,' 5 indicated 'moderate support,' and 10 denoted 'strong support.' After evaluating each PI, respondents were given the opportunity to propose modifications or to request the removal of any indicator. After the completion of the first survey round, responses from eight participants were collected and subjected to analysis.

**Table 1:** Summary of PIs and values assigned by professionals in the first survey round

Dimension	Summary of first-round. How the informants evaluated importance and relevance of indicators (scale 0–10)	Importance of indicator Average (standard deviation)	Importance of indicator Median	Relevance of indicator Average (standard deviation)	Relevance of indicator Median	Indicator selected for second-round survey
Dimension: Service availability	Percentage of answered contacts	9,10 (0,78)	9,00	8,90 (1,36)	8,50	x
	Waiting times (straight answered or callbacks)	9,00 (1,00)	9,00	8,90 (1,05)	9,00	x
	Arrival time of the phone call	8,60 (1,11)	9,00	8,30 (1,39)	8,50	
	Type of the phone call by the lines	6,60 (1,41)	7,00	6,30 (1,56)	6,00	
	Duration of the phone call	4,80 (2,11)	4,50	4,80 (2,33)	4,00	
Dimension: Patient experience	Professional qualifications and length of work experience	8,90 (1,62)	9,50	8,60 (1,73)	9,50	x
	Patient experience	8,50 (1,32)	9,00	8,30 (1,39)	8,50	x
	Professional attitude	8,30 (1,85)	9,00	7,90 (2,47)	9,00	
	Quality of communication	8,30 (1,71)	9,00	7,80 (1,39)	7,50	
	Patient satisfaction to service	8,30 (1,56)	8,50	8,30 (1,56)	8,50	
	Frequency of complaints and official reminders about the service	7,00 (1,87)	7,00	6,90 (1,54)	7,00	
Dimension: Quality and safety	The severity of patient symptoms and urgency of follow-up care needed	9,30 (0,83)	9,50	9,40 (0,86)	10,00	x
	Reason for encounter (as ICPC-2)	8,80 (1,30)	9,00	8,60 (1,41)	9,00	x
	Development of the patient's symptom/discomfort	7,80 (1,83)	7,50	7,80 (1,83)	7,50	
	Possible cause and effect of patient symptoms	7,80 (3,16)	7,00	7,60 (3,16)	7,00	
	Clinical outcomes	7,80 (1,62)	7,50	7,50 (1,20)	7,50	
	Duration of the patient's symptom	6,50 (3,16)	7,50	6,40 (3,11)	7,00	
	Safety of service	5,50 (2,00)	5,00	4,90 (1,96)	5,00	
Dimension: The outcome of the telephone triage process	The outcome of the telephone triage assessment process (type and level of the follow up contact)	8,60 (1,30)	8,50	8,60 (1,30)	8,50	x
	Review of patient Medical history	7,90 (1,96)	8,50	8,00 (1,50)	8,00	x
	Patient compliance for follow-up instructions	7,90 (1,69)	8,00	7,60 (2,00)	8,00	
	Patient medication	7,60 (2,00)	8,00	7,60 (2,06)	8,00	
	Mortality	6,40 (3,00)	6,00	6,30 (3,03)	6,00	

Using the responses gathered during the first round, we generated a summary that included the two most significant indicators evaluated for each dimension. The indicators with the highest average scores within their respective dimensions were then incorporated into the assessment framework for the second round (Table 1). The questionnaire for the second round was adjusted based on the feedback received during the initial round.

### Round two

During the second round, the participants (N = 10) appraised each PI delineated in Table 2 from three distinct perspectives, utilising a Likert scale that ranged from 1 to 10. Initially, the participants evaluated the extent to which each indicator accurately mirrored the performance dimension it purported to represent. In instances where a respondent deemed an indicator as inadequately representative, indicated by a score between 0 and 6, they were prompted to propose an alternative PI more closely aligned with the dimension. Subsequently, the indicators were assessed for their precision and sensitivity. Precision refers to an indicator's capacity to measure its intended metric accurately. Conversely, sensitivity assessment involves considering the indicator's vulnerability to external factors and the consistency of measurements obtained using the same indicator. After evaluating each dimension, respondents were invited to provide supplementary remarks.

**Table 2:** Summary of PIs and assigned values in the second survey round and selected indicators for the third round

Indicators selected for the second-round survey by dimensions	Importance of indicator (0–10 scale)		Relevance of indicator (0–10 scale)		Sensitivity of indicator (0–10 scale)		Indicator selected for third-round survey
	Average	Median	Average	Median	Average	Median	
*Dimension: Service availability*							
Percentage of answered calls	9.1	8	8.9	8	7.4	7	x
Waiting times (directly answered or callbacks)	9	8	8.9	7	7.4	7	x
*Dimension: Patient experience*							
Professional qualifications and length of work experience	8.9	7	8.6	7	5.8	5	Rejected based on second-round responses
Patient experience	8.5	8	6	7	6.4	6	**Indicator replaced in the third round:** How does the patient rate the utility of the service
*Dimension: Quality and safety*							
Severity of patient symptoms and urgency of follow-up care needed	9.3	9.5	9.4	9.5	8.2	8	x
Reason for encounter (as ICPC-2)	8.8	8	8.6	7	7.6	7	x
*Dimension: The outcome of the telephone triage process*							
Outcome of the telephone triage assessment process (type and level of the follow-up contact)	8.6	8	8.6	9	7.4	8	x
Review of patient Medical history	7.9	9	8	8	7.8	7	x
*Dimension: Costs per case*							
Production costs and total costs of patient episode	9.3	9.5	9.3	9.5	5.4	5	**Indicator replaced in the third round:** Production costs per call to the Medical Helpline 116,117
Utilization of medical Helpline service	8.1	8.5	8.1	8.5	7.4	8	

During the initial response period for the second round, only four responses were received. Additionally, three reminder emails were dispatched to encourage further participation in the survey, resulting in a total of five responses for this round.

The professionals did not advocate for the replacement of any specific indicator. However, they offered substantial critique concerning the indicator designed to gauge patient experience via the Net Promoter Score (NPS), citing its vulnerability to distortions. In addition, ‘How does the patient rate the utility of the Medical Helpline service?’ [2] was selected as a patient experience measure.

As a result, the indicator 'Professional qualifications and length of work experience' was omitted from the framework, based on the rationale that although there may be a correlation between a nurse's experience and qualifications, this parameter was deemed insufficiently robust. Furthermore, the measurement of costs was acknowledged as crucial. However, the NGT group noted the inadequacy of current reporting systems in facilitating production measurement and the comprehensive costs of the entire patient episode.

In the third round, the focus shifted to evaluating the feasibility of measuring the direct costs associated with the MH based on suggestions from experts. This approach was also seen as an indicator of the service's utilisation rate. The experts additionally highlighted the role of the telephone triage service in alleviating the growing demand and congestion in emergency departments, underscoring its significance within the acute care system.

Subsequent to the second round, a workshop was convened among the authors to meticulously analyse the responses and refine the framework in preparation for the final third round. This included integrating feedback received and considering additional aspects, such as the framework's relevance and balance (Fig. 1). Ultimately, the framework was adjudged suitable for evaluating the performance of telephone triage services.

### Round three

During the third round, respondents were tasked with evaluating the framework development as a whole based on the insights gathered in the preceding rounds. The first validation round yielded only five responses, indicating limited engagement. This outcome necessitated the conduct of a final performance framework validation round.

**Fig. 2:**
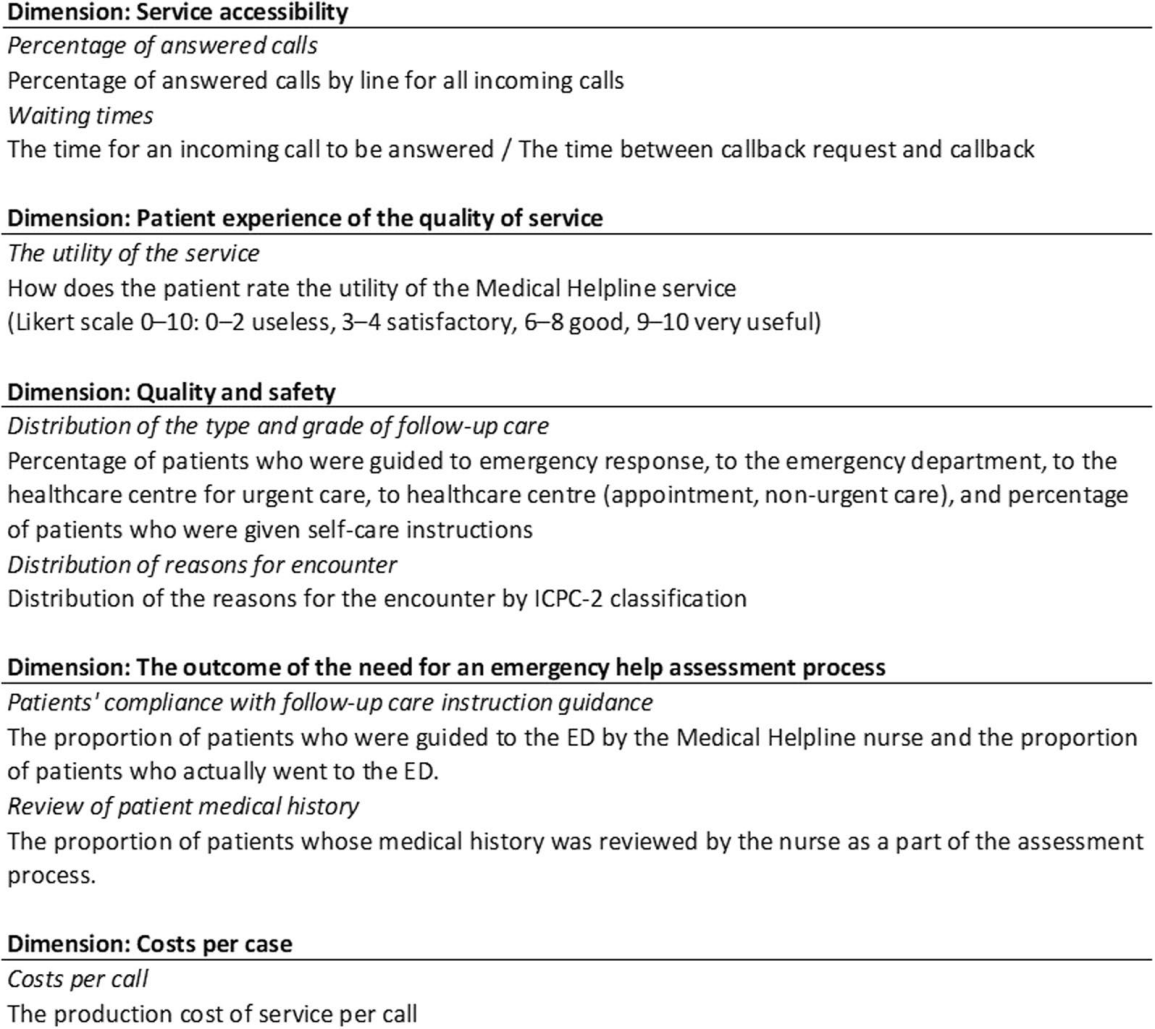
Performance measurement framework

In response to the low response rates observed during the first three rounds and to further confirm the utility of our framework, a final performance framework validation round was conducted. This round involved the participation of an expert group (*n* = 2). The evaluation criteria for these validation rounds focused on assessing the framework's contribution to the development of telephone triage services and its effectiveness in aiding both short-term and long-term strategic planning for these services. Additional considerations included the framework's relevance to the current context of telephone triage services and its overall balance. Participants were also invited to share their insights on the strengths and limitations of the framework, as well as to provide open-ended feedback for further refinement (Fig. 2).

**Fig. 3:**
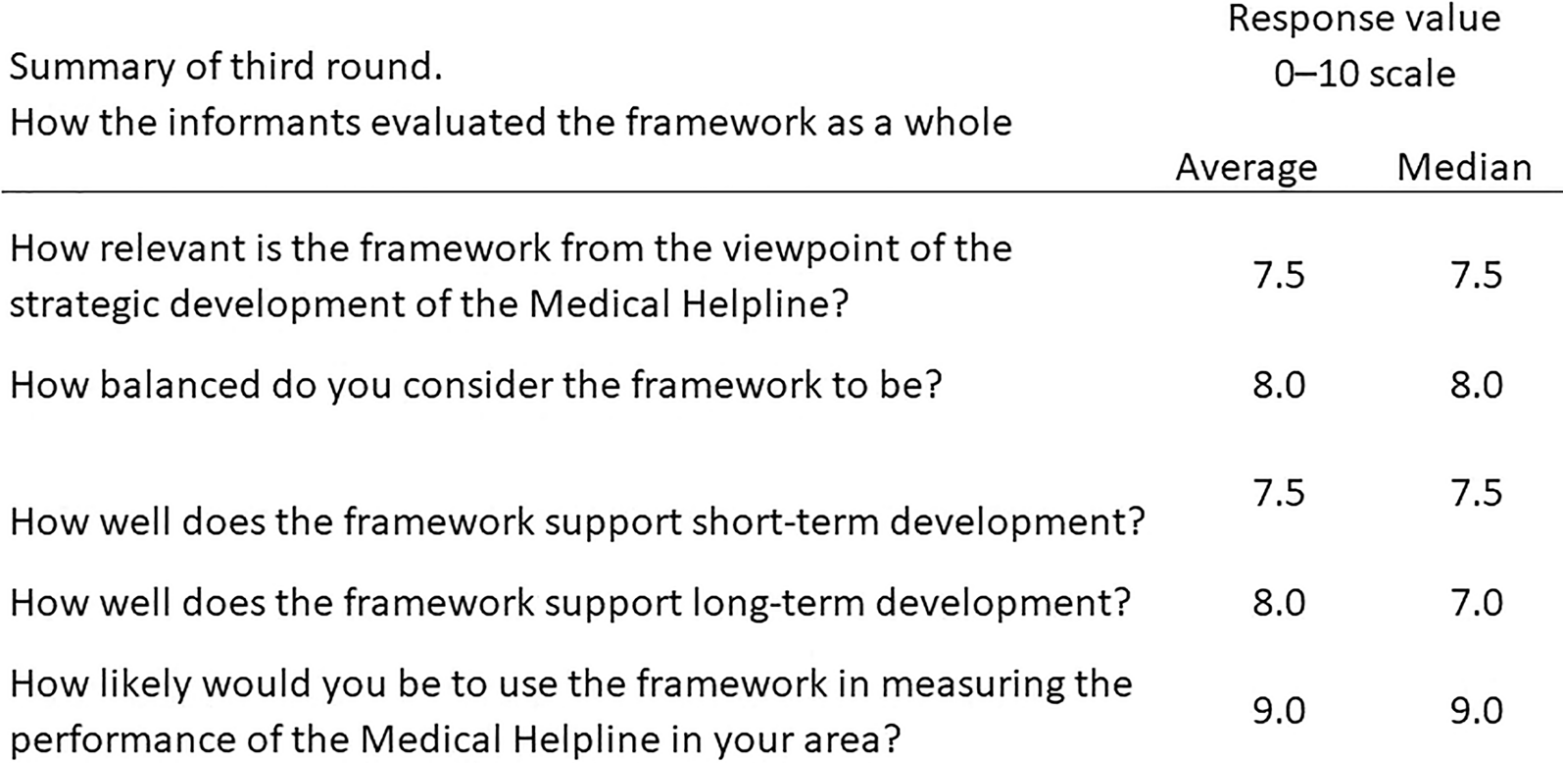
Summary of the third NGT round

In the validation rounds (Fig. 3), professionals conducted a comprehensive assessment of the framework. It was rated as moderately to highly pertinent to the strategic development of MH services, with an average score of 7.5 on a 0–10 scale. The equilibrium of the framework was acknowledged with an average score of 8.0, signalling a strong consensus on its impartial considerations across various facets of service delivery. The facilitation provided by the framework for both short-term and long-term development was similarly commended, with both dimensions garnering a score of 8.0. This score signifies confidence in its effectiveness over diverse operational timelines. Most conspicuously, the propensity of these professionals to implement this framework in gauging the performance of their respective MH was significantly favored, evidenced by a high average score of 9.0. Such a score underlines a vigorous validation of the framework's utility and supports its prospective adoption into standard practices for continuous quality evaluation.

## Discussion

In this study, our objective was to identify and rank indicators by constructing a performance measurement framework tailored for telephone triage as MH services. Despite the widespread adoption of telephone triage, there remains a lack of consensus regarding the assessment of the service's overall performance [9].

Our study proposes that performance should be evaluated using various dimensions, including access to telephone triage services, patient experiences, quality and safety, outcomes of the assessment process, and cost per case. All the PIs included have been previously discussed in the literature [2, 3, 25–40]. The professionals involved expressed their satisfaction with the measurement framework, deeming it well-balanced and supportive of strategic development in both the short and long term. Furthermore, they demonstrated a strong commitment to implementing the measurement framework in practical settings.

An effective and validated framework is crucial due to increasing societal demands on public health services for transparency, accountability, and performance [7]. In comparison to the AQTT tool developed by Graversen et al. in 2019, which focuses on measuring the quality of telephone triage, our study is distinct in that the proposed framework additionally includes indicators for measuring accessibility, patient compliance, and cost. Consequently, our study contributes to the field by providing a comprehensive framework aimed at evaluating overall performance in telephone triage. However, there is still no consensus on an effective method for evaluating health service performance nor agreement on the relevant performance dimensions [41]. As highlighted by other research, identifying appropriate PIs is essential for measuring performance. The outcome of this study is a framework comprising five dimensions and a set of eight individual PIs specifically designed to assess the performance of telephone triage services.

The findings of this study underscore the necessity for a comprehensive measurement framework that encompasses various performance dimensions. The professionals demonstrated a strong willingness to integrate the framework into their organisations, highlighting the demand for a standardised approach to evaluating the performance of telephone triage services. The results of this research could assist directors and developers in comprehending the significance of measuring overall performance as a component of an organisation's strategic development.

However, practical challenges limited the ability to incorporate all valuable PIs into the framework. For instance, while measuring the cost per patient for the entire patient episode is deemed crucial, it presents significant difficulties. These challenges are associated with the use of multiple information systems and diverse reporting practices. Consequently, it is imperative to enhance information management and standardise reporting procedures to facilitate performance measurement in practice. The responses indicated a strong consensus on PIs related to service availability, quality, and safety, as well as outcome-related indicators of telephone triage services. Implementing comprehensive performance measurement across all system levels, with suitable indicators and measures that encompass all essential performance elements, remains a challenging endeavour [42]. The organisational structure and information systems of healthcare delivery can pose obstacles to effective measurement. There has been a tendency to measure what is easily quantifiable rather than focusing on what is most impactful on outcomes [43]. Our study also revealed that some PIs, while considered valuable, were excluded due to the challenges associated with data collection in real-world settings, such as obtaining total cost per patient data.

The developed measurement framework offers a comprehensive, unified method and structure for measuring results, evaluating them, and supporting strategic management to provide information about the service's performance in relation to the established goals. The developed framework includes eight result PIs, which draw attention to the evaluation of key outcomes and create a basis for identifying the need for change and supporting decision-making.

In our ongoing study, we test the measurement tool, evaluating its usability and usefulness in practice. The renewal of organisational structures and the presence of diverse information systems pose challenges to the implementation of measurements. Despite these challenges, it is essential to critically evaluate and demonstrate the positioning of the service as part of the system. The perspectives to be evaluated include, among other things, how the new service channels affect the patient's treatment path. Regarding service availability, key questions revolve around the service itself. The widespread use of the metric system allows for the definition of the initial level of service performance and the setting of goals at the national, regional, and local levels. Consistently conducted performance measurements help identify the best and most effective improvement measures to achieve these goals and align the objectives of actors at the national level with uniform goals.

Previously, a comprehensive and established method for evaluating the effectiveness of telephone triage services was lacking. Our earlier scoping review identified that most research in this field primarily focused on health outcomes and patient experiences, offering limited perspectives [10].

To address this gap, we implemented the NGT, a consensus method, to develop a structured measurement framework aimed at seeking acceptance and publication of the framework prior to its implementation in a real-world, practical setting. This framework was designed to be broadly accepted by ED professionals. NGT was an ideal choice because of its efficiency in prioritising and reaching consensus on essential PIs for telephone triage services. This technique not only accelerated the consensus-building process but also ensured the inclusion of diverse performance dimensions related to telephone triage. A pivotal advantage of NGT was its ability to foster equitable participation among all stakeholders, which is crucial for capturing the nuances of participants' priorities. This approach is vital for guiding the effective distribution of health resources and improving service quality [13, 14].

However, this study has its limitations. The small number of participants in the NGT group means that the results may not fully represent the perspectives of a wider array of professionals on this subject. We added one additional validation round to increase the reliability of the results. Furthermore, the opportunity to conduct workshops with participants, as opposed to surveys, could have added value to the development of the framework. Another limitation lies in the study’s focus on the MH in Finland, necessitating the submission of our findings to the international community for further evaluation and validation in the future. The study also limits the building of the framework, and future studies will report the results of the use of the framework in practice.

## Conclusion

This study validated a performance measurement framework for telephone triage services, utilising existing literature and the NGT method. The framework includes five key dimensions: patient experience, quality and safety, outcome of the telephone triage process, cost per case, and eight PIs. It offers a structured and comprehensive approach to measuring the overall performance of telephone triage services, enhancing our ability to evaluate these services effectively.

### Supplementary Information

Additional file 1.

## Data Availability

Anonymised data of the NGT rounds will be available for sharing on reasonable request. Please send an email to hanna.m.vainio@helsinki.fi.

## Abbreviations

ED: Emergency department
PI: Performance indicator
NGT: Nominal group technique
MH: Medical Helpline 116,117
HER: Electronic health record
NPS: Net promoter score
